# Exploring COVID-19 conspiracy theories: education, religiosity, trust in scientists, and political orientation in 26 European countries

**DOI:** 10.1038/s41598-023-44752-w

**Published:** 2023-10-23

**Authors:** Piotr Jabkowski, Jan Domaradzki, Mariusz Baranowski

**Affiliations:** 1 Faculty of Sociology, Adam Mickiewicz University, Szamarzewkiego 89C, 60-568 Poznan, Poland; 2 Laboratory of Health Sociology and Social Pathology, Department of Social Sciences and Humanities, Poznan University of Medical Sciences, Poznan, Poland

**Keywords:** Disease prevention, Health policy

## Abstract

The COVID-19 virus disseminated globally at an accelerated pace, culminating in a worldwide pandemic; it engendered a proliferation of spurious information and a plethora of misinformation and conspiracy theories (CTs). While many factors contributing to the propensity for embracing conspiracy ideation have been delineated, the foremost determinant influencing individuals’ proclivity towards CT endorsement appears to be their level of educational attainment. This research aimed to assess the moderating effect of religiosity, trust in scientists, and political orientation on the impact of education level on people’s belief in COVID-19-related CTs in Europe by considering both individual-level and country-level contextual covariates of CT. We analysed data from the newest European Social Survey (ESS10) round conducted between September 2020 and September 2022 in 26 countries. We found religiosity weakens, and trust in scientists strengthens the effect of education, while the impact of political orientation is not straightforward. The result also demonstrates a significant negative correlation between the aggregate country-level data of the respondents supporting CTs and the level of vaccination and cumulative excess deaths in Europe. We concluded with a recommendation that planning effective public health strategies and campaigns are insufficient when based solely on people’s education, as individuals’ beliefs moderate the effect of education.

## Introduction

Conspiracy theory (CT) attributes the causes of significant social and political events to small but powerful groups of individuals or organisations that act secretly for their benefit and against the common good^1,2^. While CTs are omnipresent among Western societies, as it was shown that many members of the public endorse at least one conspiracy theory^3^, they become more prevalent in times of emergencies, crises, and uncertainty^4,5^.

Not surprisingly, ever since the coronavirus disease 2019 (COVID-19) caused by a novel severe acute respiratory syndrome coronavirus 2 (SARS-CoV-2) spread rapidly around the world from Wuhan, Hubei province of China in December 2019, becoming a global health, social and economic crisis, it leads to many CTs^6,7^. While CTs emerged shortly after the first news of COVID-19, they skyrocketed when the World Health Organisation (WHO) declared the spread of coronavirus to be a pandemic on 11 March 2020. During lockdowns, the usage of social media has increased. Such platforms as YouTube, Facebook, and Twitter have become major vectors for the dissemination of COVID-19-related CTs^8^, leading to a unique “infodemic” and general mass suspicions about what is going on^9^.

The spread of the “infodemic” has significant consequences, as CTs can affect people’s health behaviours and adherence to preventive measures during the COVID-19 pandemic. In fact, from the very beginning, when various CTs about the origins of the COVID-19 virus, the effectiveness of vaccines, and their side effects were spreading, they have been associated with people’s perception of infection risk^10,11^ and their resistance to following early control measures and non-pharmaceutical interventions which intended to curb the spread of infection, i.e. using personal protective equipment, social distancing, testing, isolation, quarantine and lockdown^12–16^. Moreover, while research showed that historically, beliefs in CTs impeded population immunisation^17^, COVID-19-related CTs were also associated with an increased tendency to resist vaccination^18–20^. Consequently, CTs have been identified as a significant threat to public health^21^.

At the same time, previous research on CTs often focused either on psychological, political, or structural aspects, suggesting that peoples’ conspiratorial thinking results either from psychological factors (i.e. biases, emotions, various personality traits, intuitive and paranoid style of thinking and “us versus them” worldviews), ideology (i.e. political and ideological self-identification, populism, institutional distrust), or having disadvantaged social status (i.e. ethnic minorities or lower SES)^1,2^. Consequently, particular attention has been paid to education^5,22–24^, religiosity^3,25–29^, political orientation^25,30,31^, and trust in science^18–20,32–35^.

Researchers consider education an essential covariate of believing in CTs, among many factors. For example, Swami et al.^24^ suggested that people with higher education are better trained in analytical or logical thinking are more aware of counterarguments, which makes them more resistant to conspiracy beliefs. Douglas et al.^23^ showed that a higher level of education reduces one’s tendency to attribute agency and intentionality where it does not exist and decreases personal appeal. Besides, van Prooijen^36^ demonstrated that people with higher education are more sceptical about the idea that complex problems can have simple solutions and have greater feelings of control of their social environment, resulting in scepticism towards CTs.

Regarding other factors impacting people’s beliefs in CTs, many studies suggested a positive correlation between religiosity and CTs. It was argued that people who declare themselves religious are more sceptical about science and tend to be more bound to conservatism and traditionalism, which offer support for conspiracy beliefs more often^3,26^. It was also shown that apocalyptic beliefs in hidden forces shaping human history can also drive support for CTs^25^. Finally, while some conspiracy narratives have quasi‐religious elements, including prophecy, esotericism, and millennialism^27^, it was suggested that not religiosity itself but somewhat dogmatic religious belief, i.e., fundamentalism, had an impact on CTs^28^.

Past studies also demonstrated that the tendency to endorse CTs is strongly influenced by the rejection or distrust of science and scepticism towards scientists^34^. While lower trust in scientists was associated with higher susceptibility to COVID-19-related misinformation^33^, higher confidence in both science and scientists was the strongest predictor of adherence to public health behaviours for fighting the pandemic^32,35^. In addition, people with higher trust in science were less likely to believe in CTs that frame vaccines as dangerous or unnecessary and were more likely to get vaccinated^18–20^.

Finally, research showed that political orientation, particularly political extremism, was associated with conspiracy thinking^23,30^. van Prooijen et al.^30^ explained it as a result of a highly structured thinking style that intends to make sense of societal events and is characterised by the belief in simplistic explanations of societal problems. Moreover, political extremism was strongly associated with dichotomous, i.e., black-and-white, thinking in which social events are classified as good or evil, like positive or negative^31^.

Inspired by previous studies, our analysis considers education an essential covariate of an individual's belief in CTs and controls for the impact of people’s ideological orientation, including religiosity, trust in scientists, and political self-placement. While previous research often linked CTs to one of these factors or included them parallelly as covariates, we aim to assess the moderating effect of education on the association between religiosity, trust in scientists, political orientation, and peoples’ support for CTs. Besides, our study's objective is to examine the extent to which individual beliefs in CT, aggregated to the country level, translate into an overall population willingness to vaccinate (i.e., vaccination rate) and the assessment of the impact of this aggregated willingness on the efficiency of the population health care system (measured by daily excess deaths per 100 k). Thus, our study’s originality lies in including individual-level determinants of people’s beliefs and country-level contextual covariates of aggregated beliefs in CTs in explaining cross-national variation in the country-level fractions of population vaccination rate, which we demonstrate also have an impact on the efficiency of public health institutions and must be included in planning the information policy.

## Methods

### Study design and data sources

Our analyses utilised data from the newest 10th round of the European Social Survey (ESS10) with a fieldwork period covering September 2020–September 2022 (all data are publicly available via the ESS Data Portal: https://ess-search.nsd.no/). A primary collection method for the ESS10 was based on a face-to-face standardised survey questionnaire. However, due to the impact of the COVID-19 pandemic, six countries switched to a self-completion (web or paper) mode of data collection, while 20 used face-to-face in-person or video interviews data gathering.

We worked on the most recent ESS10 data published in May 2023 (edition 3.0) from 26 countries with a total sample of 55,555 interviews conducted with individuals aged 15 years and older living in private households within country borders, irrespective of nationality, citizenship, language, or legal status. Note that ESS10 was also conducted in France and Montenegro; however, the question measuring beliefs in CTs (see outcome variable below) was not asked in the two countries.

Table 1 presents the list of all countries included in our analysis with their respective sample sizes and modes of data collection. We also show descriptive statistics for the outcome variable, and for two contextual country-level data we used to demonstrate how aggregated individuals’ beliefs in CTs correlate with the country-level fraction of vaccination rate and the efficiency of the national health system through cumulative excess deaths per 100 people.

**Table 1:** Descriptive characteristics of countries covered by the ESS10.

Country	Sample size	Mode of data collection	Respondents supporting CTs [in %]	People fully vaccinated per 100 people	Cumulative excess deaths per 100 k people
Austria	2003	Self-completion	16.8%	72.4	292.5
Belgium	1341	F2F	17.9%	77.5	277.3
Bulgaria	2718	F2F	55.3%	28.9	1091.3
Croatia	1592	F2F + video	39.0%	54.0	652.8
Czechia	2476	F2F	28.2%	64.3	482.1
Estonia	1542	F2F + video	27.3%	63.3	418.6
Finland	1577	F2F + video	12.0%	76.0	234.0
Germany	8725	Self-completion	15.2%	74.0	269.9
Greece	2799	F2F + video	22.0%	70.9	380.9
Hungary	1849	F2F	28.1%	60.5	510.9
Iceland	903	F2F + video	9.9%	76.1	82.0
Ireland	1770	F2F	26.0%	79.4	155.3
Israel	1308	F2F + self-completion	19.3%	64.2	142.1
Italy	2640	F2F + video	25.3%	79.2	464.8
Latvia	1023	F2F + self-completion	26.8%	66.6	597.5
Lithuania	1659	F2F	28.6%	67.2	919.7
Netherlands	1470	F2F + video	10.6%	67.2	270.0
North Macedonia	1429	F2F + video	53.6%	38.4	836.4
Norway	1411	F2F + video	10.3%	73.3	138.4
Poland	2065	Self-completion	25.4%	55.2	495.3
Portugal	1838	F2F + video	31.6%	85.1	358.6
Serbia	1505	Self-completion	38.5%	46.0	920.2
Slovakia	1418	F2F	37.4%	45.1	570.4
Slovenia	1252	F2F	35.1%	56.0	325.4
Spain	2283	Self-completion	29.6%	81.3	330.7
Sweden	2287	Self-completion	8.1%	70.3	172.8
Switzerland	1523	F2F + video	17.3%	67.6	240.2
United Kingdom	1149	F2F + video	21.9%	72.1	301.3

face-to-face interviews (F2F).

### Outcome variable

*COVID-19 conspiracy theory beliefs*. The interviewers asked respondents whether they agree that “coronavirus is the result of deliberate and concealed efforts of some government or organisations” on a 5-point scale: 1 (agree strongly), 2 (agree), 3 (neither agree nor disagree), 4 (disagree), 5 (disagree strongly). For analysis, we dichotomised the outcome variable such that respondents who agree or strongly agree with the statement were assigned code 1 and the rest code 0. From analysis, we excluded respondents who indicated “Do not know” or “Refusal” because both options are treated in the ESS10 as item-nonresponse cases.

### Covariates variables

*The level of education* was measured by implementing the International Standard Classification of Education (ISCED). We created the variable *level of education* with values as follows: 1 (ISCED IV-VI; reference category), 2 (ISCED III), 3 (ISCED II), and 4 (ISCED I).

*Religiosity*. Respondents were asked to indicate how religious they are on an 11-point scale ranging from 0 (Not at all religious) to 10 (Very religious). In the regression analysis, we standardised *religiosity* by calculating z-scores across countries and respondents.

*Trust in scientists*. Respondents assessed whether they trust scientists on an 11-point scale ranging from 0 (No trust at all) to 10 (Complete trust); note that the ESS10 does not ask for trust in science. Original scale points were standardised across all countries and respondents before including the variable in regression analysis. Note that the question was omitted in Czechia and Estonia; thus, we excluded two countries from the regression.

*Political Left–Right*. Political orientation was measured by asking respondents to position themselves along a left–right 11-point rating scale ranging from 1 (left) to 10 (right). Again, we standardised the original scale points by calculating z-scores across all countries and respondents.

### Demographics

Sex was indicated as 0 (woman) and 1 (man), while age—expressed in the number of years—was divided by 10 to avoid small numbers in the regression coefficient estimates.

### Country-level contextual data

We also included two contextual variables to test the relationship between the overall fraction of respondents supporting the COVID-19-related CTs in respective countries and a) the number of people fully vaccinated per 100 citizens and b) cumulative excess deaths per 100,000 citizens. Both a) and b) were derived from the publicly available dataset on COVID-19 vaccinations (https://github.com/owid/covid-19-data/tree/master/public/data/vaccinations) and excess deaths (https://github.com/TheEconomist/covid-19-the-economist-global-excess-deaths-model). The reference date for contextual data was set up to the end of 2022 and corresponded to the end of the year when the ESS10 fieldwork was finished.

### Data analysis

To recognise the hierarchical structure of ESS10 data with respondents nested within countries and to test the effects of all explanatory variables, demographics, and the relationship between contextual country-level data and the aggregate values of the outcome variable (i.e., the overall fraction of respondents believing in CTs), we implemented multilevel logistic regressions (assuming random intercepts between countries) with all variables added to the subsequent models step-by-step:

- The null model (with no predictors) allows us to estimate the variance components, i.e., the intraclass correlation coefficient (ICC) attributed to the country level.
- Model 1.1. adds the number of people fully vaccinated per 100 citizens as a country-level contextual variable.
- Model 1.2. adds the cumulative excess deaths per 100,000 citizens as a country-level covariate.

Note in models 1.1 and 1.2. we added country-level variables separately instead of including them in one regression, as the small number of countries in our comparison increases the risk of overfitting, and the statistical procedures might have limited power to detect significant effects when both country-level variables are incorporated.

- Model 2 adds education, religiosity, trust in scientists, political orientation, sex, and age to the variables included in Model 1.1. and 1.2.
- Finally, models 3.1–3.3 add interactions between the level of education and (3.1) religiosity, (3.2) trust in scientists, and (3.3) political orientation, respectively.

Models 3.1–3.3 check whether the effect of education is moderated by religiosity, trust in scientists, and political orientation. In these models, we allowed the impact of respective moderators to vary between countries (i.e., we estimated random slope models).

In addition, to check whether the observed effects of education, religiosity, trust in scientists, and political orientation are robust on the model specification and do not depend on the specificity of the multilevel approach, we implemented four by-country regressions (separately for each covariate) on 26 different subsets from each country. In by-country regression, we included sex, age, and added education, religiosity, trust in scientists, and political orientation, respectively. Supplementary Online Materials (hereafter, SOM) contains the detailed specification of multilevel and by-country models. All analyses were performed in the R Project for Statistical Computing, and for data analyses and visualisations, we implemented the R packages listed in SOM.

### Ethical approval

This study exclusively used publicly available aggregate data sets and published research, and hence no ethics approval was required.

## Results

We started with descriptive analyses comparing 26 countries. Then, we moved to multilevel and by-country regressions to examine the contribution of each predictor while adjusting for country-level variation in the outcome variable. Finally, we analysed the relation between the overall country fraction of individuals believing in CTs and the vaccination rate, as well as the efficiency of the national health care system.

### Individual-level covariates of respondents’ beliefs in CTs

We started with an empty model, with the country as a nesting variable for respondents, to check whether notable differences between countries in the outcome variable exist and need to be accounted for. The ICC value indicates that 9.5 per cent of total variation can be attributed to differences between countries; thus, the multilevel approach is justified. By including both country-level contextual variables (see Table 2), namely, the number of people fully vaccinated per 100 citizens and the cumulative daily excess deaths per 100 k people, we reduced unexplained cross-country variance to 5.7 and 2.6 per cent, respectively. What is essential is that both country-level predictors are significant. This result stands in line with the descriptive results.

**Table 2:** Individual-level covariates of CTs: regression results as odds ratios (OR) and standard errors (SE) of OR in perenties.

Predictors	Null model	Model 1.1	Model 1.2
Intercept	0.301*** (0.036)	2.275 (1.008)	0.301*** (0.019)
People fully vaccinated per 100		0.970*** (0.006)	
Cumulative daily excess deaths per 100 k			1.219*** (0.030)
ICC	0.095	0.057	0.026
N of countries	26	26	26
N of respondents	41,367	41,367	41,367
Marginal R^2^ / Conditional R^2^	0.000/ 0.095	0.048/0.102	0.070/0.095
AIC	25,733.4	25,721.0	25,706.1
log-Likelihood	− 12,864.7	− 12,857.5	− 12,850.1

*p < 0.05; **p < 0.01; ***p < 0.001.

Table 3 summarises the multilevel logistic regressions with individual-level covariates included in the models of increased complexity. Note that the number of people fully vaccinated per 100 citizens allows us to explain cross-national differences a little worse compared to the cumulative daily excess deaths per 100 k people (see Table 2); thus, we present the results for regressions with the latter country-level variable included. Note that we also transformed regression coefficients into standardised odds ratios using the natural exponential function to facilitate the interpretation of the effects.

**Table 3:** Individual-level covariates of CTs: regression results as odds ratios (OR) and standard errors (SE) of OR in perenties.

Predictors	Model 2	Model 3.1	Model 3.2	Model 3.3
Intercept	0.212*** (0.016)	0.211*** (0.016)	0.217 *** (0.016)	0.217 *** (0.016)
Gender [Female = 1]	0.871 *** (0.026)	0.875 *** (0.026)	0.867 *** (0.026)	0.873 *** (0.026)
Age	1.006 (0.008)	1.007 (0.008)	1.003 (0.008)	1.003 (0.008)
ISCED III [vs. VI-V]	1.544 *** (0.057)	1.538 *** (0.057)	1.553 *** (0.058)	1.5339 *** (0.058)
ISCED II [vs. VI-V]	1.799 *** (0.074)	1.799 *** (0.074)	1.809 *** (0.076)	1.842 *** (0.077)
ISCED I [vs. VI-V]	2.136 *** (0.121)	2.196 *** (0.127)	2.246 *** (0.127)	2.168 *** (0.124)
Cumulative daily excess deaths per 100 k	1.232 *** (0.016)	1.230 *** (0.030)	1.254 *** (0.022)	1.293 *** (0.029)
Religiosity (z-values)	1.051 ** (0.016)	1.189 *** (0.036)	1.049 ** (0.016)	1049 ** (0.016)
Trust in scientists (z-values)	0.673 *** (0.010)	0.675 *** (0.010)	0.559 *** (0.025)	0.677 *** (0.010)
Political Left–Right (z-values)	1.284 *** (0.019)	1.282 *** (0.019)	1.279 *** (0.019)	1.321 *** (0.047)
ISCED III * Religiosity		0.868 *** (0.032)		
ISCED II * Religiosity		0.844 *** (0.035)		
ISCED I * Religiosity		0.833 *** (0.045)		
ISCED III * Trust in scientists			1.205 *** (0.044)	
ISCED II * Trust in scientists			1.245 *** (0.049)	
ISCED I * Trust in scientists			1.327 *** (0.065)	
ISCED III * Political Left–Right				0.928 * (0.035)
ISCED II * Political Left–Right				0.858 *** (0.035)
ISCED I * Political Left–Right				0.890 * (0.047)
ICC	0.028	0.029	0.037	0.030
N of countries	26	26	26	26
N of respondents	41,367	41,367	41,367	41,367
Marginal R^2^ / Conditional R^2^	0.159/0.182	0.161/0.185	0.183/0.213	0.187/0.212
AIC	24,272.1	24,255.1	24,108.1	24,246.2
log-Likelihood	− 12,125.0	− 12,111.5	− 12,038.1	− 12,107.1

*p < 0.05; **p < 0.01; ***p < 0.001.

At the respondent’s level, our results confirm previous findings indicating the significant effect of education (belief in CTs declines as education increases). We also found a significant positive effect of religiosity (probability for supporting CTs increases with religiosity), a negative effect of trust in scientists (more confidence, less support for CTs), and a positive effect of political orientation, with right-wing people more supporting CTs.

This result reveals several significant findings concerning individual-level determinants of CT belief. Firstly, our results validate previous research by affirming the substantial impact of education on CT beliefs, demonstrating a clear and statistically significant decline in CT endorsement as education levels rise. Furthermore, we identified a significant positive effect of religiosity, indicating that greater religiosity is associated with an increased likelihood of supporting CTs. Conversely, trust in scientists exhibited a negative effect, demonstrating that individuals with higher trust in scientific expertise are less inclined to support CTs. Lastly, political orientation emerged as a significant factor, with right-wing individuals displaying a higher propensity to endorse CTs.

**Figure 1:**
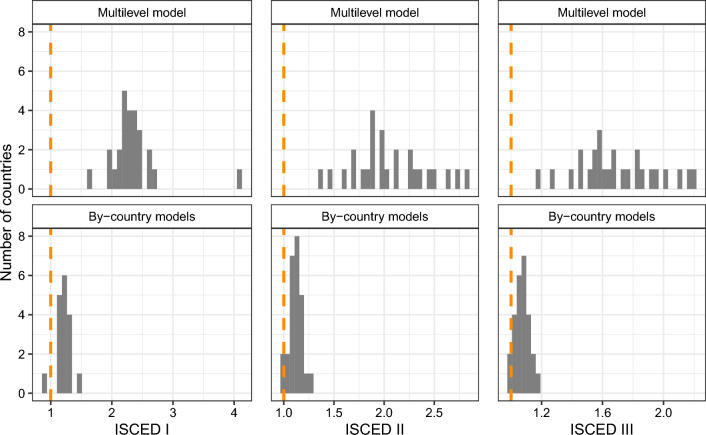
Multilevel and by-country estimates of country slopes (odds ratios) for education.

**Figure 2:**
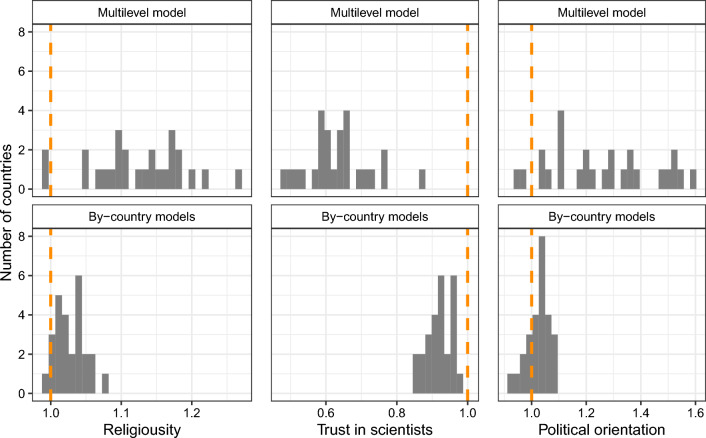
Multilevel and by-country estimates of country slopes (odds ratios) for religiosity, trust in scientists, and political orientation.

The effects of education, religiosity, trust in scientists, and political orientation are in the same direction in most European countries. In Fig. 1, we compare the distribution of regression coefficients for the education derived from multilevel models (upper panel) with the distribution from by-country analyses (lower panel), while in Fig. 2, the distribution of country slopes for religiosity, trust in scientists, and political orientation.

Starting from the results presented in Fig. 1, it is essential to note that in all countries, the reference category for the three educational levels is ISCED IV-VI, for which the odds ratio is fixed and equal to 1.0. As we can note, in the case of multilevel models, for all countries (and in by-country models in almost all countries), the odds ratios are above the reference value 1.0, which demonstrates that the pattern of relationship between individuals’ educational level and their belief in CTs is common across Europe.

For the religiosity and political orientation (see Fig. 2), in the case of multilevel models, all country slopes, except two, are above the reference value of 1.0. For trust in scientists, all are below. In the by-country models, almost all slopes are above 1.0 for religiosity and political orientation, while for all countries, odds ratios are below 1.0 for trust in scientists. Such distributions of odds ratios are less likely under the assumption of no assumed directions of the relationship between belief in CTs and individuals’ religiosity, trust in scientists, and political orientation.

**Figure 3:**
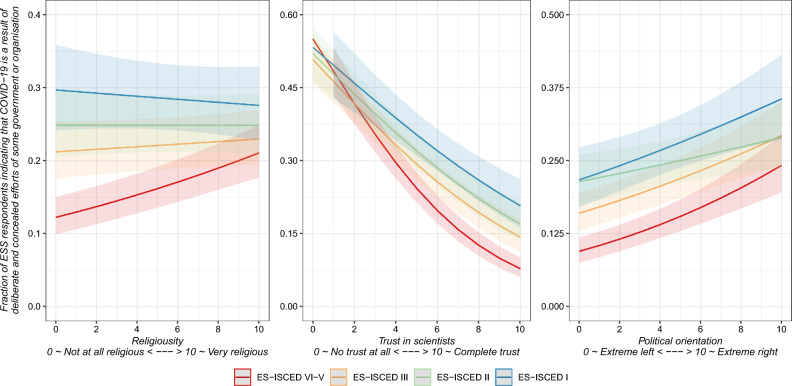
Moderating effect of religiosity, trust in scientists, and political orientation on the impact of the level of education on CT.

In the last part of our analysis, we will nuance our exploration of individual-level patterns of respondents’ support for CT, and we will focus on how religiosity, trust in scientists, and political orientation moderate the relationship between the level of education and the likelihood of supporting CTs. By exploring how these factors interact with education, we contextualise the influence of education and recognise that its impact may vary across individuals with different levels of religiosity, trust in scientists, or political orientation. This analysis has practical implications for tailoring interventions to specific subgroups. It offers visual representations to simplify the interpretation of complex interactions, ultimately contributing to our comprehension of the drivers of CT beliefs and their broader societal implications. As previously mentioned, all three interactions were positively tested (see models 3.1–3.2 in Table 2), and to simplify the interpretation of the moderating effects, we plotted the marginal effects of interactions in Fig. 3.

Starting from the left panel of our analysis, we observe an interplay between religiosity and education, unveiling how these factors influence CT beliefs. It becomes evident that religiosity has a moderating effect and weakens the impact of education on one's susceptibility to CTs. Among respondents who identify as religious, the influence of educational attainment appears to be less pronounced than their non-religious counterparts. This result suggests that, for religious persons, higher levels of education may not substantially decrease belief in CTs as it does for those with a secular worldview.

Moving to the middle panel, trust in scientists emerges as a factor that bolsters education's influence on belief in CTs. Individuals who trust scientists tend to be more receptive to the insights and knowledge gained through education, thus reinforcing the counter-conspiratorial effect of learning. This phenomenon starkly contrasts the situation of those who do not trust scientists. For this group, the impact of education takes an unexpected turn, deviating from the general trend. Instead of education diminishing belief in CTs, we find that highly educated individuals who lack trust in scientists tend to embrace conspiracy theories to a greater extent than their less-educated counterparts.

Turning to the right panel, which delves into the realm of political orientation, the moderating effect of political orientation on the relationship between education and belief in CTs appears less straightforward as only for respondents with the level of education on ISCED II the effect of political orientation is weakened. In this context, the impact of one's political leanings on susceptibility to CTs seems less pronounced, suggesting that for individuals at this specific educational threshold, other unmeasured factors may play a more prominent role in shaping their beliefs.

### Individual’s beliefs in CTs impact the vaccination rate at the country level and the efficiency of the national healthcare system

Now, we move to the analysis focusing on how aggregated individual beliefs in COVID-19-related CTs impact the vaccination rate at the population level and the efficiency of the national healthcare system.

**Figure 4:**
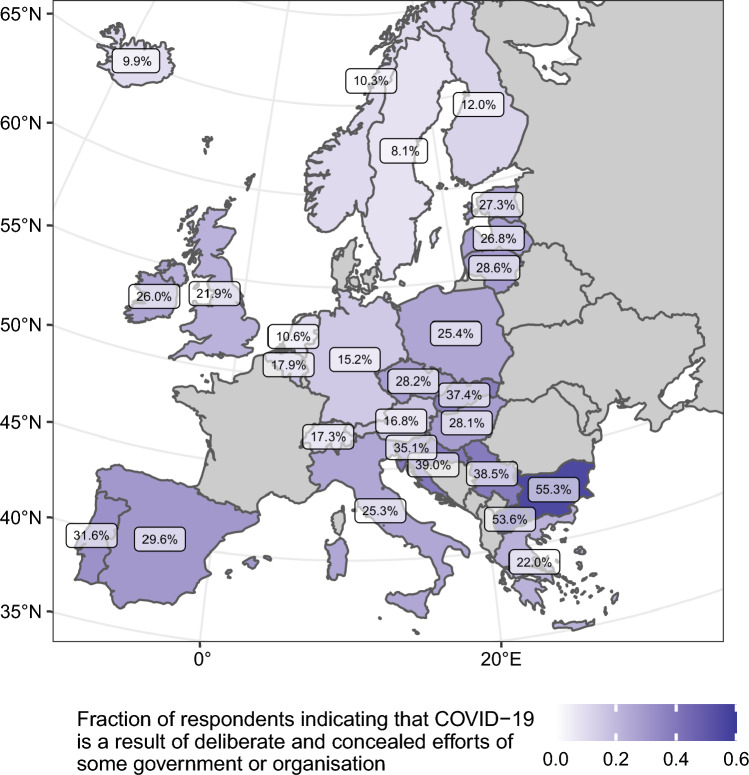
Cross-country differences in the fraction of respondents indicate that COVID-19 is a result of deliberate and concealed efforts of some government or organisations.

Figure 4 shows a map of Europe with a visual overview of the aggregated support for CTs at the country level across countries, with notable cross-country differences between European regions, with Central and Eastern Europe (CEE) and the Balkan countries showing higher levels of belief in CTs. The ranking is opened by two outlying countries, i.e., Bulgaria and North Macedonia (with more than 50 per cent of respondents indicating that COID-19 is the result of deliberate and hidden efforts by some governments and organisations) and closed by all Northern countries, which show the lowest overall support for CTs among all countries covered by the ESS Round 10. In the discussion section of our paper, we attribute these differences to, among others, the generally higher levels of trust in government and public institutions observed in the Northern and Western countries, with the Balkan, Southern, and CEE countries showing lower levels of trust.

**Figure 5:**
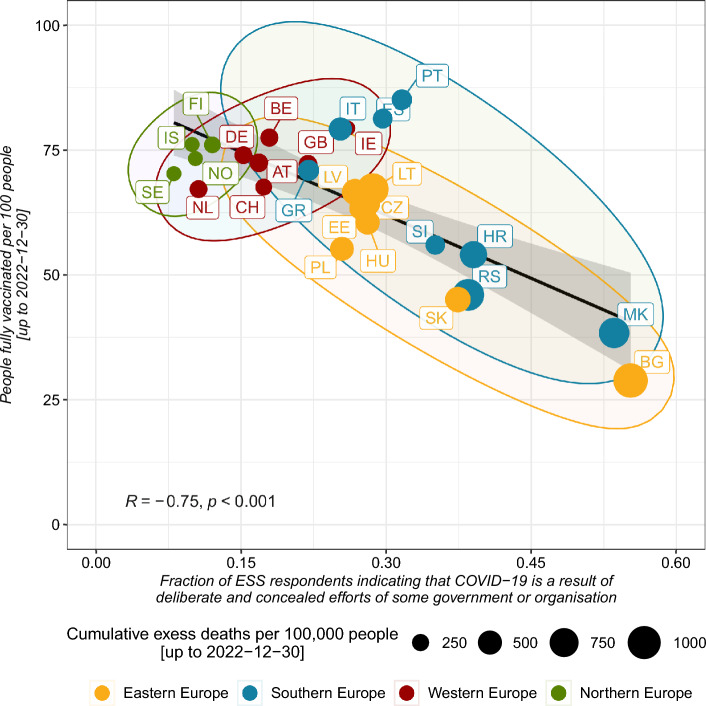
Country-level association between outcome and contextual variables.

The cross-national variations in the prevalence of individuals endorsing COVID-19-related CTs are clearly visible in our analysis; however, our study aims to explore how these individual beliefs about CTs, when aggregated to the country level, translate into overall population willingness to vaccinate and how this may affect the efficiency of the population's health care system. We also scrutinise if any such dissimilarities exist in European regions. Figure 5 displays the correlation between the fraction of respondents supporting CTs at the country level (x-axis) and the proportion of people who are fully vaccinated (y-axis), with the cumulative excess deaths per 100,000 people (represented by the size of the dots). Each dot on the x- and y-axis represents a country, with the colour indicating the European region. Through correlational analysis, it is evident that in countries with a higher prevalence of CTs, the effectiveness of vaccination programs is lower, and the healthcare system efficiency is compromised, as shown by the excess deaths per 100,000 people. A notable observation is a distinct bi-polarisation between the CEE and Balkan countries versus the rest of Europe.

## Discussion

Since the outbreak of the COVID-19 pandemic, governments across Europe intended to curb the transmission of the novel coronavirus by implementing multiple public health interventions and control measures. Simultaneously, during the past few years, CTs have continued to diffuse in many Western democracies, leading to many protest movements against the policy measures that aimed at containing the diffusion of coronavirus^7,12,32,35^. The conclusion is alarming since CTs undermine trust in public health institutions and programs and reduce pandemic preparedness and compliance with government guidelines that can limit the spread of infections and increase vaccination^19,20^.

Understanding the factors that promote and undermine the spread and belief in CTs is becoming a key challenge for public health policy to develop solutions for preventing and monitoring health and epidemic risks. Our analysis corresponds to the findings of previous research conducted at the individual level^23^, as we found that the level of education plays an essential role in shaping people’s belief in COVID-19-related CTs in Europe. However, contrary to other studies, we proved that the correlation between educational level and willingness to believe in CTs is not fixed among all individuals. In particular, we found that religiosity, trust in scientists, and political orientation of respondents moderate the negative impact of the level of education on people’s conspiracy beliefs. As a key message, our findings imply that public health policies should be tailored to different demographic groups, strategically allocate resources, and engage in effective communication strategies to address conspiracy beliefs' complex and evolving nature. Such an approach, informed by data-driven decision-making and interdisciplinary collaboration, can enhance the efficiency of public policy in preventing and monitoring health and epidemic risks while promoting trust in scientific authorities.

An undoubted advantage of our research is, in fact, the inclusion of 26 European countries, enabling us to articulate cross-country variations in the level of supporting CTs. This cross-country perspective goes beyond the single-country focus in many previous studies^12–14^, which does not allow for a more holistic view of national differences. Thus, our study demonstrated how the individual threats associated with CTs translate into public health risks for whole populations to many officials and scientists. The elevated support for CTs within CEE and Balkan nations, as illustrated in Fig. 5, can be attributed to multiple causal factors reflective of the historical socialist legacy in this region. Primarily, a salient factor is the diminished confidence level in governmental bodies and institutional entities, precipitated by a hesitance to unquestioningly embrace the official government perspective. This scepticism subsequently engenders a proclivity among the populace to subscribe to alternative narratives, manifesting as the propagation and acceptance of CTs^37,38^.

Consequently, a correlation exists between this phenomenon and social polarisation^39,40^, thereby engendering a predisposition towards repudiating established information outlets, thus fostering a conducive milieu for the proliferation of CTs. Acknowledging the pivotal role played by socioeconomic determinants, which significantly contribute to socioeconomic disparities and perceived injustices, further reinforces the fertile ground upon which CTs thrive. As a key message of our study, policymakers should consider the importance of acknowledging and addressing the unique socio-cultural and historical factors that contribute to the propagation of CTs in some countries, and public health efforts and policies should be tailored to the country enhancing their efficiency in countering the spread of CTs and mitigating their associated public health risks. In conclusion, additional work using historical specificities and patterns of political culture (particularly in CEE and Balkans) is needed to fully understand the dynamics of the processes of creation and dissemination of conspiracy concepts central to the health security of entire societies.

Although our study includes two levels of analysis (individual—regarding respondent’s characteristics and country—regarding vaccination rate and excess deaths), it is not without limitations. The former relates to the covariates, other than education level, religiosity, trust in scientists, and political orientation, that could be included to explain why people believe in CTs. Since our choice was justified by a specific research objective and derived from a literature review, these additional variables could shed a different light on the issue of public attitudes towards COVID-19-related conspiracy beliefs. Futhermore, an attempt to build comprehensive policy recommendations for the healthcare system based on our analysis's results may be challenging due to the complex character of individual motivations and subjective components of CT covariates. Finally, although the results showed the dissimilarity of CEE and the Balkan countries from the rest of Europe regarding coronavirus vaccination and healthcare system efficiency, we could not explain this key difference based on the ESS data alone. To do so, we used the results of other studies that only indirectly addressed the question of the reasons for regional differences concerning beliefs in CTs, vaccination rate, and excess deaths per 100,000 citizens.

## Conclusion

During a burgeoning body of research dedicated to exploring vaccination and CTs within the backdrop of the COVID-19 crisis, our analytical endeavour seeks to transcend the confines of exclusively individual or aggregate explanations pertaining to the aforementioned issue. Our study explored the covariates of conspiracy theories at the individual and country levels. At the individual level, we found that education significantly influenced people's beliefs in COVID-19-related CTs. However, this relationship was subject to moderation by factors such as religiosity, trust in scientists, and political orientation. In turn, on the country level, our analysis revealed significant cross-country variation between Northern and Western Europe on the one side, and CEE and Balkan states on the other, in CT beliefs with strict implications for vaccination rate and efficiency of the healthcare system during the COVID-19 pandemic. Combining these two levels provides a holistic perspective, enabling policymakers and researchers to develop targeted and nuanced approaches to mitigate the impact of CTs on public health.

While this study recognises that education is among the key factors fuelling individuals’ support for CTs, it shows that cultural, social, and political contexts play a crucial role in vaccine acceptance and decision-making. Consequently, it suggests that although it is often argued that to fight CTs and vaccine hesitancy successfully, social policy and vaccination campaigns should address the “knowledge deficit” and focus on advancing peoples’ health or biotechnological literacy, educational programs may not be as efficient as one could hope, since there is no simple relationship between education and CTs support. Thus, by stressing the moderating role of religiosity, trust in scientists, and political orientation, this study’s results can help to understand that social policy and vaccination campaigns should involve several sectors of society and require a long-term effort from various stakeholders. Simultaneously, since this is one of the few studies that assessed the prevalence and determinants of CTs simultaneously in 26 European countries, it highlights the importance of addressing the factors mentioned above at the individual and the country level. By showing that when aggregated at the country level, these individual-level determinants impact the overall population’s willingness to vaccinate against health threats, this study can help understand how factors influencing people’s conspiracy beliefs can affect acceptance of public health policies during the crisis.

### Supplementary Information

Supplementary Information.

## Data Availability

All data are publicly available via the ESS Data Portal: https://ess-search.nsd.no/.
