# Supplementary material for: Exploring COVID-19 conspiracy theories: education, religiosity, trust in scientists, and political orientation in 26 European countries
Source: Sci Rep. 2023 Oct 23;13:18116. doi: 10.1038/s41598-023-44752-w (PMC10593806; doi:10.1038/s41598-023-44752-w)
Supplement: Supplementary file 1 — Supplementary Information. [file 41598_2023_44752_MOESM1_ESM.docx]

Supplementary material: Exploring COVID-19 Conspiracy Theories: Education, Religiosity, Trust in Scientists, and Political Orientation in 26 European Countries

**Table of content**

[A. Software for data manipulation and analysis 1](#_Toc147143447)

[B. Replication of R codes for data manipulation and producing all results 1](#_Toc147143448)

[B.1. Data manipulation 1](#_Toc147143449)

[B.2. R codes for replication Table 1 4](#_Toc147143450)

[B.3. R codes for specification of multilevel regressions 6](#_Toc147143451)

[B.4. R codes for replication Table 2 8](#_Toc147143452)

[B.5. R codes for replication Table 3 9](#_Toc147143453)

[B.6. R codes for replication Figure 1 (including specification of by-country models) 9](#_Toc147143454)

[B.7. R codes for replication Figure 2 (including specification of by-country models) 12](#_Toc147143455)

[B.8. R codes for replication Figure 3 14](#_Toc147143456)

[B.9. R codes for replication Figure 4 16](#_Toc147143457)

[B.10. R codes for replication Figure 5 17](#_Toc147143458)

[References 19](#_Toc147143459)

# **A. Software for data manipulation and analysis**

We performed all analyses in the R Project for Statistical Computing (1). For data manipulation, descriptive studies, regressions, and data visualizations, we implemented the following R packages: tidyverse (2); haven (3); flextable (4); ggplot2 (5); lme4 (6); sjPlot (7); broom (8); sf (9); rnaturalearth (10); rnaturalearthdata (11); rgeos (12).

# **B. Replication of R codes for data manipulation and producing all results**

## **B.1. Data manipulation**

library(tidyverse)
library(haven)
library(flextable)
library(ggplot2)
library(lme4)
library(sjPlot)
library(broom)
# maps
library(sf)
library(rnaturalearth)
library(rnaturalearthdata)
library(rgeos)

# load ESS data

data_ESS10 <- read_sav("ESS-Data-Wizard-subset-2023-05-12.sav") %>%
 rowid_to_column() %>%
 mutate(#Calculate weight for analysis as a product of population size weights and post-stratification weights
 anweight = pspwght * pweight,
 #Dependent variable
 gvconc19_recoded = ifelse(gvconc19 == 1 | gvconc19 == 2, 1, ifelse(is.na(gvconc19), NA_real_, 0)),
 #Socio-demographic characteristics
 gndr = ifelse(gndr == 1, "Male", "Female"),
 gndr = fct_relevel(gndr, c("Male", "Female")),
 age = (agea - mean(agea, na.rm = TRUE))/10) %>%
 #Socio-economic characteristics
 mutate(eisced = case_when(eisced == 1 ~ "ES-ISCED I",
 eisced == 2 ~ "ES-ISCED II",
 eisced == 3 ~ "ES-ISCED III",
 eisced == 4 ~ "ES-ISCED III",
 eisced == 5 ~ "ES-ISCED VI-V",
 eisced == 6 ~ "ES-ISCED VI-V",
 eisced == 7 ~ "ES-ISCED VI-V",
 eisced == 55 ~ NA_character_,
 is.na(eisced) ~ NA_character_),
 eisced = fct_relevel(eisced, "ES-ISCED VI-V", "ES-ISCED III", "ES-ISCED II", "ES-ISCED I"),
 # health = case_when(health == 1 ~ "Very good",
 # health == 2 ~ "Good",
 # health == 3 ~ "Poor",
 # health == 4 ~ "Poor",
 # health == 5 ~ "Poor",
 # is.na(health) ~ NA_character_),
 # health = fct_relevel(health, c("Very good", "Good", "Poor"))
 )

lrscale_z_values <- data_ESS10 %>%
 group_by(cntry) %>%
 filter(!is.na(lrscale)) %>%
 mutate(lrscale_z_values = as.vector(corpcor::wt.scale(lrscale, w = anweight))) %>%
 ungroup() %>%
 select(rowid, lrscale_z_values)

rlgdgr_z_values <- data_ESS10 %>%
 group_by(cntry) %>%
 filter(!is.na(rlgdgr)) %>%
 mutate(rlgdgr_z_values = as.vector(corpcor::wt.scale(rlgdgr, w = anweight))) %>%
 ungroup() %>%
 select(rowid, rlgdgr_z_values)

trstsci_z_values <- data_ESS10 %>%
 group_by(cntry) %>%
 filter(!is.na(trstsci)) %>%
 mutate(trstsci_z_values = as.vector(corpcor::wt.scale(trstsci, w = anweight))) %>%
 ungroup() %>%
 select(rowid, trstsci_z_values)

generalised_trust_z_values <- data_ESS10 %>%
 group_by(cntry) %>%
 filter(!is.na(ppltrst), !is.na(pplfair), !is.na(pplhlp)) %>%
 rowwise() %>%
 mutate(generalised_trust = mean(ppltrst, pplfair, pplhlp)) %>%
 ungroup() %>%
 group_by(cntry) %>%
 mutate(generalised_trust_z_values = as.vector(corpcor::wt.scale(generalised_trust, w = anweight))) %>%
 ungroup() %>%
 select(rowid, generalised_trust_z_values)

data_ESS10_for_analysis <- data_ESS10 %>%
 left_join(., rlgdgr_z_values, by = "rowid") %>%
 left_join(., lrscale_z_values, by = "rowid") %>%
 left_join(., trstsci_z_values, by = "rowid") %>%
 left_join(., generalised_trust_z_values, by = "rowid")

# load data on share of people fully vaccinated - covid

countries_ESS10 <- data_ESS10_for_analysis %>%
 count(cntry) %>%
 mutate(cntry = countrycode::countrycode(tolower(cntry), origin = "iso2c", destination = "iso3c")) %>%
 pull(cntry)

modes_of_data_collection <- read.csv("Modes_of_data_collection.csv") %>%
 as_tibble() %>%
 mutate(cntry = countrycode::countrycode(tolower(cntry_name), origin = "country.name", destination = "iso2c"))

data_vaccination_covid <- read.csv("share-people-fully-vaccinated-covid.csv") %>%
 as_tibble() %>%
 filter(Code %in% countries_ESS10) %>%
 mutate(Day = as.Date(Day)) %>%
 filter(Day >= "2021-10-01" & Day <= "2022-12-30") %>%
 group_by(Code) %>%
 summarise(people_fully_vaccinated_per_hundred = mean(people_fully_vaccinated_per_hundred)) %>%
 rename(cntry = Code) %>%
 mutate(cntry = countrycode::countrycode(tolower(cntry), origin = "iso3c", destination = "iso2c"))

excess_deaths_cumulative_per_100k_economist <- read.csv("excess-deaths-cumulative-per-100k-economist.csv") %>%
 as_tibble() %>%
 filter(Code %in% countries_ESS10) %>%
 mutate(Day = as.Date(Day)) %>%
 filter(Day == "2022-12-26") %>%
 rename(cntry = Code) %>%
 mutate(cntry = countrycode::countrycode(tolower(cntry), origin = "iso3c", destination = "iso2c"),
 cumulative_estimated_daily_excess_deaths_per_100k_centred = (cumulative_estimated_daily_excess_deaths_per_100k - mean(cumulative_estimated_daily_excess_deaths_per_100k))/100)

data_ESS10_for_analysis_merged_with_country_level <- data_ESS10_for_analysis %>%
 left_join(., modes_of_data_collection, by = "cntry") %>%
 left_join(., data_vaccination_covid, by = "cntry") %>%
 left_join(., excess_deaths_cumulative_per_100k_economist, by = "cntry") %>%
 select(cntry, anweight, region, gvconc19_recoded, gvconc19, gndr, age, eisced, health,
 rlgdgr_z_values, lrscale_z_values, trstsci_z_values, rlgdgr, lrscale, trstsci, mode.y,
 people_fully_vaccinated_per_hundred, cumulative_estimated_daily_excess_deaths_per_100k, cumulative_estimated_daily_excess_deaths_per_100k_centred) %>%
 rename(mode = mode.y)

# ESS countries with missing gvconc19 & trstsci

cntry_missing <- data_ESS10_for_analysis_merged_with_country_level %>%
 group_by(cntry) %>%
 mutate(x = ifelse(is.na(gvconc19), 1, 0),
 y = ifelse(is.na(trstsci), 1, 0)) %>%
 summarise(mean_x = mean(x),
 mean_y = mean(y)) %>%
 filter(mean_x == 1 | mean_y == 1) %>%
 zap_formats() %>% zap_label() %>%
 zap_labels() %>%
 pull(cntry)

cntry_included <- data_ESS10_for_analysis_merged_with_country_level %>%
 group_by(cntry) %>%
 mutate(x = ifelse(is.na(gvconc19), 1, 0),
 y = ifelse(is.na(trstsci), 1, 0)) %>%
 summarise(mean_x = mean(x),
 mean_y = mean(y)) %>%
 filter(mean_x != 1 & mean_y != 1) %>%
 zap_formats() %>% zap_label() %>%
 zap_labels() %>%
 pull(cntry)

cntry_included_plots <- c(cntry_included, "CZ", "EE")

## **B.2. R codes for replication Table 1**

# Table 1. Description of the sample

table1_1 <- data_ESS10_for_analysis_merged_with_country_level %>%
 filter(cntry %in% cntry_included_plots) %>%
 mutate(Country = countrycode::countrycode(tolower(cntry),
 origin = "iso2c",
 destination = "country.name")) %>%
 group_by(Country) %>%
 count() %>%
 select(Country, n) %>%
 rename("Sample size" = n)

table1_2 <- data_ESS10_for_analysis_merged_with_country_level %>%
 filter(cntry %in% cntry_included_plots) %>%
 mutate(Country = countrycode::countrycode(tolower(cntry),
 origin = "iso2c",
 destination = "country.name")) %>%
 group_by(Country) %>%
 count(mode) %>%
 select(-n) %>%
 rename("Mode of data collection" = mode)

table1_3 <- data_ESS10_for_analysis_merged_with_country_level %>%
 filter(cntry %in% cntry_included_plots) %>%
 filter(!is.na(gvconc19)) %>%
 mutate(gvconc19 = ifelse(gvconc19 == 1 | gvconc19 == 2, 1, 0)) %>%
 rowwise() %>%
 mutate(gvconc19 = gvconc19 * anweight) %>%
 ungroup() %>%
 mutate(Country = countrycode::countrycode(tolower(cntry),
 origin = "iso2c",
 destination = "country.name")) %>%
 group_by(Country) %>%
 summarise(mean_gvconc19 = sum(gvconc19)/sum(anweight)) %>%
 mutate(fraction = paste0(format(round(mean_gvconc19 * 100, digits = 1), nsmall = 1), "%")) %>%
 select(Country, fraction) %>%
 rename(`Fraction of respondets\nsupporting COVID-19 related\nconspiracy teory` = fraction)

table1_4 <- data_ESS10_for_analysis_merged_with_country_level %>%
 filter(cntry %in% cntry_included_plots) %>%
 mutate(Country = countrycode::countrycode(tolower(cntry),
 origin = "iso2c",
 destination = "country.name")) %>%
 group_by(Country) %>%
 summarise(`People fully vaccinated\nper 100 people\n[up to 2022-12-30]` = format(round(mean(people_fully_vaccinated_per_hundred), digits = 1), nsmall=1))

table1_5 <- data_ESS10_for_analysis_merged_with_country_level %>%
 filter(cntry %in% cntry_included_plots) %>%
 mutate(Country = countrycode::countrycode(tolower(cntry),
 origin = "iso2c",
 destination = "country.name")) %>%
 group_by(Country) %>%
 summarise(`Cumulative exess deaths\nper 100,000 people\n[up to 2022-12-30]` = format(round(mean(cumulative_estimated_daily_excess_deaths_per_100k), digits = 1), nsmall=1))

Table1 <- left_join(table1_1, table1_2, by = "Country") %>%
 left_join(., table1_3, by = "Country") %>%
 left_join(., table1_4, by = "Country") %>%
 left_join(., table1_5, by = "Country") %>%
 qflextable() %>%
 bold(bold = TRUE, part = "header") %>%
 align(j = 2:5, align = c("center"), part = "header") %>%
 align(j = 2:5, align = c("center"), part = "body") %>%
 fontsize(size = 10, part = "all")

Table1

## **B.3. R codes for specification of multilevel regressions**

#Regression

 # Multilevel regression models (respondents nested within countries)

 Data_for_regression <- data_ESS10_for_analysis_merged_with_country_level %>%
 zap_label() %>% zap_labels() %>%
 filter(!is.na(cntry), !is.na(anweight),
 !is.na(gvconc19_recoded),
 !is.na(gndr), !is.na(age),
 !is.na(eisced),
 !is.na(health),
 !is.na(trstsci_z_values),
 !is.na(rlgdgr_z_values),
 !is.na(lrscale_z_values),
 !is.na(cumulative_estimated_daily_excess_deaths_per_100k_centred),
 !is.na(people_fully_vaccinated_per_hundred))

 # ESS countries with missing gvconc19 & trstsci

 cntry_missing <- data_ESS10 %>%
 group_by(cntry) %>%
 mutate(x = ifelse(is.na(gvconc19), 1, 0),
 y = ifelse(is.na(trstsci), 1, 0)) %>%
 summarise(mean_x = mean(x),
 mean_y = mean(y)) %>%
 filter(mean_x == 1 | mean_y == 1) %>%
 zap_formats() %>% zap_label() %>%
 zap_labels() %>%
 pull(cntry)

 # Null-model

 model0 <- glmer(gvconc19_recoded ~ 1 +
 (1 | cntry),
 weights = anweight,
 data = Data_for_regression,
 family= binomial(link = "logit"), nAGQ=0)

 # Model 1

model1a <- glmer(gvconc19_recoded ~ 1 +
 people_fully_vaccinated_per_hundred +
 (1 | cntry),
 weights = anweight,
 data = Data_for_regression,
 family= binomial(link = "logit"), nAGQ=0)

model1b <- glmer(gvconc19_recoded ~ 1 +
 cumulative_estimated_daily_excess_deaths_per_100k_centred +
 (1 | cntry),
 weights = anweight,
 data = Data_for_regression,
 family= binomial(link = "logit"), nAGQ=0)

#model1 <- glmer(gvconc19_recoded ~ 1 +
# people_fully_vaccinated_per_hundred +
# cumulative_estimated_daily_excess_deaths_per_100k_centred +
# (1 | cntry),
# weights = anweight,
# data = Data_for_regression,
# family= binomial(link = "logit"), nAGQ=0)


 # Model 2

 model2 <- glmer(gvconc19_recoded ~ 1 +
 cumulative_estimated_daily_excess_deaths_per_100k_centred +
 gndr + age +
 eisced +
 rlgdgr_z_values + trstsci_z_values + lrscale_z_values +

 (1 | cntry),
 weights = anweight,
 data = Data_for_regression,
 family= binomial(link = "logit"), nAGQ=0)

 # Models 3

 model3.1 <- glmer(gvconc19_recoded ~ 1 +
 cumulative_estimated_daily_excess_deaths_per_100k_centred +
 gndr + age +
 eisced +
 rlgdgr_z_values + trstsci_z_values + lrscale_z_values +
 rlgdgr_z_values * eisced +
 (rlgdgr_z_values | cntry),
 weights = anweight,
 data = Data_for_regression,
 family= binomial(link = "logit"), nAGQ=0)

 model3.2 <- glmer(gvconc19_recoded ~ 1 +
 cumulative_estimated_daily_excess_deaths_per_100k_centred +
 gndr + age + eisced +
 rlgdgr_z_values + trstsci_z_values + lrscale_z_values +
 eisced * trstsci_z_values +

 (trstsci_z_values | cntry),
 weights = anweight,
 data = Data_for_regression,
 family= binomial(link = "logit"), nAGQ=0)

 model3.3 <- glmer(gvconc19_recoded ~ 1 +
 cumulative_estimated_daily_excess_deaths_per_100k_centred +
 gndr + age + eisced +
 rlgdgr_z_values + trstsci_z_values + lrscale_z_values +
 eisced * lrscale_z_values +

 (lrscale_z_values | cntry),
 weights = anweight,
 data = Data_for_regression,
 family= binomial(link = "logit"), nAGQ=0)

## **B.4. R codes for replication Table 2**

Table2 <- tab_model(model0, model1a, model1b,
 p.style = "stars",
 p.threshold = c(0.05, 0.01, 0.001),
 digits = 3,
 digits.re = 3,
 transform = "exp",
 show.est = TRUE,
 show.ci = FALSE,
 show.se = TRUE,
 show.aic = TRUE,
 show.loglik = TRUE,
 show.re.var = FALSE,
 show.icc = TRUE,
 show.r2 = TRUE,
 pred.labels = c("Intercept",
 "People fully vaccinated per 100",
 "Cummulative daily excess deaths per 100k"),
 dv.labels = c("Null model",
 "Model 1.1",
 "Model 1.2"))

Table2

## **B.5. R codes for replication Table 3**

Table3 <- tab_model(model2, model3.1, model3.2, model3.3,
 p.style = "stars",
 p.threshold = c(0.05, 0.01, 0.001),
 digits = 3,
 digits.re = 3,
 transform = "exp",
 show.est = TRUE,
 show.ci = FALSE,
 show.se = TRUE,
 show.aic = TRUE,
 show.loglik = TRUE,
 show.re.var = FALSE,
 show.icc = TRUE,
 show.r2 = TRUE,
 pred.labels = c("Intercept",
 "Cummulative daily excess deaths per 100k",
 "Gender [Female = 1]",
 "Age",
 "ISCED III [vs. VI-V]",
 "ISCED II [vs. VI-V]",
 "ISCED I [vs. VI-V]",
 "Religiousity (z-values)",
 "Trust in scientists (z-values)",
 "Political Left-Right (z-values)",
 "ISCED III * Religiousity",
 "ISCED II * Religiousity",
 "ISCED I * Religiousity",
 "ISCED III * Trust in scientists",
 "ISCED II * Trust in scientists",
 "ISCED I * Trust in scientists",
 "ISCED III * Political Left-Right",
 "ISCED II * Political Left-Right",
 "ISCED I * Political Left-Right"),
 dv.labels = c("Model 2",
 "Model 3.1",
 "Model 3.2",
 "Model 3.3"))

Table3

## **B.6. R codes for replication Figure 1 (including specification of by-country models)**

# Cross-country effects

# education

tab_education_multilevel <- coef(glmer(gvconc19_recoded ~ 1 +
 gndr + age +
 eisced +
 (eisced | cntry),
 weights = anweight,
 data = Data_for_regression,
 family= binomial(link = "logit"), nAGQ=0))$cntry %>% data.frame() %>%
 tibble::rownames_to_column() %>%
 select(rowname,
 `ISCED I` = `eiscedES.ISCED.I`,
 `ISCED II` = `eiscedES.ISCED.II`,
 `ISCED III` = `eiscedES.ISCED.III`) %>%
 pivot_longer(cols = !rowname, names_to = "ISCED", values_to = "estimate") %>%
 mutate(or = exp(estimate), model = "Multilevel model")

tab_education_by_country <- Data_for_regression %>%
 nest(data = -cntry) %>%
 mutate(
 fit = map(data, ~ lm(gvconc19_recoded ~ 1 +
 gndr + age + eisced, weight = anweight, data = .x)),
 tidied = map(fit, tidy)
 ) %>%
 unnest(tidied) %>%
 filter(term == "eiscedES-ISCED III" | term == "eiscedES-ISCED II" | term == "eiscedES-ISCED I") %>%
 mutate(term = case_when(term == "eiscedES-ISCED III" ~ "ISCED III",
 term == "eiscedES-ISCED II" ~ "ISCED II",
 term == "eiscedES-ISCED I" ~ "ISCED I")) %>%
 select(rowname = cntry, ISCED = term, estimate) %>%
 mutate(or = exp(estimate), model = "By-country models")


fig_coefs_ISCED1 <- bind_rows(tab_education_multilevel %>% filter(ISCED == "ISCED I"), tab_education_by_country %>% filter(ISCED == "ISCED I")) %>%
 mutate(model = factor(model, levels = c("Multilevel model", "By-country models"))) %>%
 ggplot(., aes(x = or)) +
 geom_histogram(fill = "gray50", bins = 40) +
 geom_vline(xintercept = 1, col = "darkorange", size = 1, linetype = "dashed") +
 scale_y_continuous(breaks = seq(0 , 8, by = 2),
 limits = c(0, 8)) +
 # scale_x_continuous(breaks = c(0, 0.125, 0.25)) +
 xlab("ISCED I") +
 ylab("Number of countries") +
 theme_bw() +
 facet_wrap("model", nrow = 2) +
 theme(strip.background = element_rect(fill = "transparent"))

fig_coefs_ISCED2 <- bind_rows(tab_education_multilevel %>% filter(ISCED == "ISCED II"), tab_education_by_country %>% filter(ISCED == "ISCED II")) %>%
 mutate(model = factor(model, levels = c("Multilevel model", "By-country models"))) %>%
 ggplot(., aes(x = or)) +
 geom_histogram(fill = "gray50", bins = 40) +
 geom_vline(xintercept = 1, col = "darkorange", size = 1, linetype = "dashed") +
 scale_y_continuous(breaks = seq(0 , 8, by = 2),
 limits = c(0, 8)) +
 # scale_x_continuous(breaks = c(0, 0.125, 0.25)) +
 xlab("ISCED II") +
 ylab("Number of countries") +
 theme_bw() +
 facet_wrap("model", nrow = 2) +
 theme(strip.background = element_rect(fill = "transparent"),
 axis.title.y = element_blank(),
 axis.text.y = element_blank(),
 axis.ticks.y = element_blank())

fig_coefs_ISCED3 <- bind_rows(tab_education_multilevel %>% filter(ISCED == "ISCED III"), tab_education_by_country %>% filter(ISCED == "ISCED III")) %>%
 mutate(model = factor(model, levels = c("Multilevel model", "By-country models"))) %>%
 ggplot(., aes(x = or)) +
 geom_histogram(fill = "gray50", bins = 40) +
 geom_vline(xintercept = 1, col = "darkorange", size = 1, linetype = "dashed") +
 scale_y_continuous(breaks = seq(0 , 8, by = 2),
 limits = c(0, 8)) +
 # scale_x_continuous(breaks = c(0, 0.125, 0.25)) +
 xlab("ISCED III") +
 ylab("Number of countries") +
 theme_bw() +
 facet_wrap("model", nrow = 2) +
 theme(strip.background = element_rect(fill = "transparent"),
 axis.title.y = element_blank(),
 axis.text.y = element_blank(),
 axis.ticks.y = element_blank())


Figure_slopes_ISCED <- ggpubr::ggarrange(fig_coefs_ISCED1, fig_coefs_ISCED2, fig_coefs_ISCED3,
 ncol = 3,
 widths = c(1, 0.85, 0.85))
Figure_slopes_ISCED

Figure3 <- ggpubr::ggarrange(fig_time_coefs_religiousity, fig_time_coefs_trust_scientists, fig_time_coefs_left_right,
 ncol = 3,
 widths = c(1, 0.85, 0.85))
Figure3

## **B.7. R codes for replication Figure 2 (including specification of by-country models)**

# Cross-country effects

# religiousity
tab_religiousity_multilevel <- coef(glmer(gvconc19_recoded ~ 1 +
 gndr + age +
 eisced +
 rlgdgr_z_values +
 (rlgdgr_z_values | cntry),
 weights = anweight,
 data = Data_for_regression,
 family= binomial(link = "logit"), nAGQ=0))$cntry %>% data.frame() %>%
 tibble::rownames_to_column() %>%
 select(rowname, estimate = rlgdgr_z_values) %>%
 mutate(or = exp(estimate), model = "Multilevel model")

tab_religiousity_by_country <- Data_for_regression %>%
 nest(data = -cntry) %>%
 mutate(
 fit = map(data, ~ lm(gvconc19_recoded ~ 1 +
 gndr + age +
 rlgdgr_z_values, weight = anweight, data = .x)),
 tidied = map(fit, tidy)
 ) %>%
 unnest(tidied) %>%
 filter(term == "rlgdgr_z_values") %>%
 select(rowname = cntry, estimate) %>%
 mutate(or = exp(estimate), model = "By-country models")


fig_time_coefs_religiousity <- bind_rows(tab_religiousity_multilevel, tab_religiousity_by_country) %>%
 mutate(model = factor(model, levels = c("Multilevel model", "By-country models"))) %>%
 ggplot(., aes(x = or)) +
 geom_histogram(fill = "gray50", bins = 30) +
 geom_vline(xintercept = 1, col = "darkorange", size = 1, linetype = "dashed") +
 scale_y_continuous(breaks = seq(0 , 8, by = 2),
 limits = c(0, 8)) +
 # scale_x_continuous(breaks = c(0, 0.125, 0.25)) +
 xlab("Religiousity") +
 ylab("Number of countries") +
 theme_bw() +
 facet_wrap("model", nrow = 2) +
 theme(strip.background = element_rect(fill = "transparent"))

# trust_scientists

tab_trust_scientists_multilevel <- coef(glmer(gvconc19_recoded ~ 1 +
 gndr + age +
 trstsci_z_values +
 (trstsci_z_values | cntry),
 weights = anweight,
 data = Data_for_regression,
 family= binomial(link = "logit"), nAGQ=0))$cntry %>% data.frame() %>%
 tibble::rownames_to_column() %>%
 select(rowname, estimate = trstsci_z_values) %>%
 mutate(or = exp(estimate), model = "Multilevel model")

tab_trust_scientists_by_country <- Data_for_regression %>%
 nest(data = -cntry) %>%
 mutate(
 fit = map(data, ~ lm(gvconc19_recoded ~ 1 +
 gndr + age +
 trstsci_z_values, weight = anweight, data = .x)),
 tidied = map(fit, tidy)
 ) %>%
 unnest(tidied) %>%
 filter(term == "trstsci_z_values") %>%
 select(rowname = cntry, estimate) %>%
 mutate(or = exp(estimate), model = "By-country models")

fig_time_coefs_trust_scientists <- bind_rows(tab_trust_scientists_multilevel, tab_trust_scientists_by_country) %>%
 mutate(model = factor(model, levels = c("Multilevel model", "By-country models"))) %>%
 ggplot(., aes(x = or)) +
 geom_histogram(fill = "gray50", bins = 30) +
 geom_vline(xintercept = 1, col = "darkorange", size = 1, linetype = "dashed") +
 scale_y_continuous(breaks = seq(0 , 8, by = 2),
 limits = c(0, 8)) +
# scale_x_continuous(breaks = c(-0.9, -0.6, -0.3, 0)) +
 xlab("Trust in scientists") +
 theme_bw() +
 facet_wrap("model", nrow = 2) +
 theme(strip.background = element_rect(fill = "transparent"),
 axis.title.y = element_blank(),
 axis.text.y = element_blank(),
 axis.ticks.y = element_blank())

# left_right

tab_left_right_multilevel <- coef(glmer(gvconc19_recoded ~ 1 +
 gndr + age +
 lrscale_z_values +
 (lrscale_z_values | cntry),
 weights = anweight,
 data = Data_for_regression,
 family= binomial(link = "logit"), nAGQ=0))$cntry %>% data.frame() %>%
 tibble::rownames_to_column() %>%
 select(rowname, estimate = lrscale_z_values) %>%
 mutate(or = exp(estimate), model = "Multilevel model")

tab_left_right_by_country <- Data_for_regression %>%
 nest(data = -cntry) %>%
 mutate(
 fit = map(data, ~ lm(gvconc19_recoded ~ 1 +
 gndr + age +
 lrscale_z_values, weight = anweight, data = .x)),
 tidied = map(fit, tidy)
 ) %>%
 unnest(tidied) %>%
 filter(term == "lrscale_z_values") %>%
 select(rowname = cntry, estimate) %>%
 mutate(or = exp(estimate), model = "By-country models")

fig_time_coefs_left_right <- bind_rows(tab_left_right_multilevel, tab_left_right_by_country) %>%
 mutate(model = factor(model, levels = c("Multilevel model", "By-country models"))) %>%
 ggplot(., aes(x = or)) +
 geom_histogram(fill = "gray50", bins = 30) +
 geom_vline(xintercept = 1, col = "darkorange", size = 1, linetype = "dashed") +
 scale_y_continuous(breaks = seq(0 , 8, by = 2),
 limits = c(0, 8)) +
 # scale_x_continuous(breaks = c(-0.1, 0, 0.25, 0.5)) +
 xlab("Political orientation") +
 theme_bw() +
 facet_wrap("model", nrow = 2) +
 theme(strip.background = element_rect(fill = "transparent"),
 axis.title.y = element_blank(),
 axis.text.y = element_blank(),
 axis.ticks.y = element_blank())

# Figure 4. Country associations between a) religiosity, b) trust in scientists, c) political orientation and believing in conspiracy theory

Figure_slopes <- ggpubr::ggarrange(fig_time_coefs_religiousity, fig_time_coefs_trust_scientists, fig_time_coefs_left_right,
 ncol = 3,
 widths = c(1, 0.85, 0.85))
Figure_slopes

## **B.8. R codes for replication Figure 3**

#Plotting interactions

 # Models 3bis for making interaction plot easy to interpret

 model3.1_bis <- glmer(gvconc19_recoded ~ 1 +
 gndr + age + eisced +
 rlgdgr + trstsci + lrscale +
 rlgdgr * eisced +
 people_fully_vaccinated_per_hundred +
 (rlgdgr | cntry),
 weights = anweight,
 data = Data_for_regression,
 family= binomial(link = "logit"), nAGQ=0)

 model3.2_bis <- glmer(gvconc19_recoded ~ 1 +
 gndr + age + eisced +
 rlgdgr + trstsci + lrscale +
 eisced * trstsci +
 people_fully_vaccinated_per_hundred +
 (trstsci | cntry),
 weights = anweight,
 data = Data_for_regression,
 family= binomial(link = "logit"), nAGQ=0)

 model3.3_bis <- glmer(gvconc19_recoded ~ 1 +
 gndr + age + eisced +
 rlgdgr + trstsci + lrscale +
 eisced * lrscale +
 people_fully_vaccinated_per_hundred +
 (lrscale | cntry),
 weights = anweight,
 data = Data_for_regression,
 family= binomial(link = "logit"), nAGQ=0)

Fig_int_religiousity <- plot_model(model3.1_bis, type = "pred", terms = c("rlgdgr[all]", "eisced")) +
 labs(x = "Religiousity\n0 ~ Not at all religious < --- > 10 ~ Very religious",
 y = "Fraction of ESS respondents indicating that COVID-19 is a result of\ndeliberate and concealed efforts of some government or organisation") +
 scale_x_continuous(breaks = seq(0, 10, by = 2)) +
 scale_y_continuous(breaks = seq(0, .4, by = 0.1), limits = c(0, .4)) +
 scale_color_brewer(palette = "Spectral") +
 scale_fill_brewer(palette = "Spectral") +
 theme_bw() +
 theme(legend.position = "bottom",
 legend.title = element_blank(),
 axis.title = element_text(face = "italic", size = 10),
 plot.title = element_blank())

Fig_int_trust_scientists <- plot_model(model3.2_bis, type = "pred", terms = c("trstsci[all]", "eisced")) +
 labs(x = "Trust in scientists\n0 ~ No trust at all < --- > 10 ~ Complete trust",
 y = "Fraction of ESS respondents indicating that COVID-19 is a result of\ndeliberate and concealed efforts of some government or organisation") +
 scale_x_continuous(breaks = seq(0, 10, by = 2)) +
 scale_y_continuous(breaks = seq(0, .6, by = 0.15), limits = c(0, .6)) +
 scale_color_brewer(palette = "Spectral") +
 scale_fill_brewer(palette = "Spectral") +
 theme_bw() +
 theme(legend.position = "bottom",
 legend.title = element_blank(),
 axis.title.y = element_blank(),
 axis.title = element_text(face = "italic", size = 10),
 plot.title = element_blank())

Fig_int_left_right <- plot_model(model3.3_bis, type = "pred", terms = c("lrscale[all]", "eisced")) +
 labs(x = "Political orientation\n0 ~ Extreme left < --- > 10 ~ Extreme right",
 y = "Fraction of ESS respondents indicating that COVID-19 is a result of\ndeliberate and concealed efforts of some government or organisation") +
 scale_x_continuous(breaks = seq(0, 10, by = 2)) +
 scale_y_continuous(breaks = seq(0, .5, by = 0.125), limits = c(0, .5)) +
 scale_color_brewer(palette = "Spectral") +
 scale_fill_brewer(palette = "Spectral") +
 theme_bw() +
 theme(legend.position = "bottom",
 legend.title = element_blank(),
 axis.title.y = element_blank(),
 axis.title = element_text(face = "italic", size = 10),
 plot.title = element_blank())

#Figure 3. Interactions - religiosity, trust in scientists and political orientation moderates the impact of education on believing in conspiracy theory

Figure_interactions <- ggpubr::ggarrange(Fig_int_religiousity, Fig_int_trust_scientists, Fig_int_left_right,
 nrow = 1,
 common.legend = TRUE,
 widths = c(1, 0.95, 0.95),
 legend = "bottom")

Figure_interactions

## **B.9. R codes for replication Figure 4**

world <- ne_countries(scale = "medium", returnclass = "sf")

 Data_maps <- data_ESS10 %>%
 filter(!is.na(gvconc19)) %>%
 mutate(gvconc19 = ifelse(gvconc19 == 1 | gvconc19 == 2, 1, 0)) %>%
 rowwise() %>%
 mutate(gvconc19 = gvconc19 * anweight) %>%
 ungroup() %>%
 group_by(cntry) %>%
 summarise(mean_gvconc19 = sum(gvconc19)/sum(anweight)) %>%
 mutate(fraction = paste0(format(round(mean_gvconc19 * 100, digits = 1), nsmall = 1), "%")) %>%
 mutate(cntry = countrycode::countrycode(tolower(cntry), origin = "iso2c", destination = "iso3c"))

 Europe <- world[which(world$continent == "Europe"),]

 Europe <- st_transform(Europe, crs = 3035)

 Europe_GPS <- Europe %>% left_join(Data_maps, by = c("adm0_a3" = "cntry"))

 Figure4 <- ggplot(Europe_GPS) +
 geom_sf(aes(fill = mean_gvconc19)) +
 geom_sf_label(aes(label = fraction), fill = "white", size = 2, alpha = 0.6, fun.geometry = sf::st_centroid) +
 coord_sf(xlim = c(2500000, 6600000), ylim = c(1380000, 5300000), expand = FALSE) +
 scale_fill_gradient2(na.value = "gray80",
 limits = c(0,.6), breaks = c(0, .2, .4, .6)) +
 guides(fill = guide_colorbar(title = "Fraction of respondents indicating that COVID-19\nis a result of deliberate and concealed efforts of\nsome government or organisation",
 label.position = "bottom", ticks.linewidth = 2))+
 theme_bw() +
 theme(legend.position = "bottom",
 axis.title = element_blank(),
 legend.text = element_text(size = 9))

 Figure4

## **B.10. R codes for replication Figure 5**

Data_fig5 <- data_ESS10_for_analysis %>%
 filter(!is.na(gvconc19)) %>%
 mutate(gvconc19 = ifelse(gvconc19 == 1 | gvconc19 == 2, 1, 0)) %>%
 rowwise() %>%
 mutate(gvconc19 = gvconc19 * anweight) %>%
 ungroup() %>%
 group_by(cntry) %>%
 summarise(mean_gvconc19 = sum(gvconc19)/sum(anweight)) %>%
 mutate(fraction = paste0(format(round(mean_gvconc19 * 100, digits = 1), nsmall = 1), "%"))

data_merged <- left_join(Data_fig5, data_vaccination_covid, by = "cntry") %>%
 left_join(., excess_deaths_cumulative_per_100k_economist, by = "cntry")

 Figure5 <- data_merged %>%
 filter(cntry != "IL") %>%
 mutate(region = countrycode::countrycode(cntry,
 origin = "iso2c",
 destination = "region23"),
 region = ifelse(cntry == "CY" | cntry == "TR", "Southern Europe", region),
 region = ifelse(cntry == "LT" | cntry == "LV" | cntry == "EE", "Eastern Europe", region),
 region = ifelse(cntry == "IE" | cntry == "GB", "Western Europe", region),
 region = factor(region, levels = c("Eastern Europe", "Southern Europe", "Western Europe", "Northern Europe"))) %>%
 ggplot(aes(x=mean_gvconc19, y = people_fully_vaccinated_per_hundred)) +
 geom_smooth(method = 'lm', colour = "black") +
 ggforce::geom_mark_ellipse(aes(color = region, fill = region),
 alpha = 0.05,
 expand = unit(1,"cm")) +
 ggrepel::geom_label_repel(aes(label=cntry, color= region), alpha = 0.9) +
 ggpubr::stat_cor(color = "grey10",
 p.accuracy = 0.001,
 r.accuracy = 0.01,
 label.x.npc = 0.05,
 label.y.npc = 0.05) +
 geom_point(aes(color = region), size = 4) +
 geom_point(aes(color = region, size = cumulative_estimated_daily_excess_deaths_per_100k)) +
 scale_size(range = c(1,10)) +
 scale_color_manual(values = c("#FAAB18", "#1380A1","#990000", "#588300")) +
 scale_y_continuous(breaks = seq(0, 100, by = 25), limits = c(0, 100)) +
 scale_x_continuous(breaks = seq(0, .6, by = .15), limits = c(0, .60)) +
 scale_color_manual(values = c("#FAAB18", "#1380A1","#990000", "#588300")) +
 labs(x="Fraction of ESS respondents indicating that COVID-19 is a result of\ndeliberate and concealed efforts of some government or organisation",
 y="People fully vaccinated per 100 people\n[up to 2022-12-30]",
 color = "",
 fill = "",
 size = "Cumulative exess deaths per 100,000 people\n[up to 2022-12-30]") +
 theme_bw() +
 theme(legend.position="bottom", legend.box="vertical", legend.margin=margin(),
 legend.text = element_text(size=10),
 legend.title = element_text(hjust = 0.5),
 axis.text = element_text(size=10, color="black"),
 axis.title = element_text(size=10, face = "italic"),
 plot.title = element_text(hjust = 0.5, face = "bold", size = 10))
 Figure5

# **References**

1. R_Core_Team. R: A language and environment for statistical computing. R Foundation for statistical computing, Vienna. 2021.

2. Wickham H, Averick M, Bryan J, Chang W, McGowan LDA, François R, et al. Welcome to the Tidyverse. Journal of open source software. 2019;4(43):1686.

3. Wickham H, Miller E. haven: Import and Export 'SPSS', 'Stata' and 'SAS' Files. 2022.

4. Gohel D. flextable: Functions for Tabular Reporting. R package version 0.6.9. 2021.

5. Wickham H. ggplot2: Elegant Graphics for Data Analysis. New York: Springer Cham; 2016.

6. Bates D, Mächler M, Bolker B, Walker S. Fitting Linear Mixed-Effects Models Using lme4. Journal of Statistical Software. 2015;67(1):1 - 48.

7. Lüdecke D. sjPlot: Data visualization for statistics in social science. R package version 2.8.10. 2021.

8. Robinson D, Hayes A, Couch S. broom: Convert Statistical Objects into Tidy Tibbles. 2022.

9. Pebesma E. Simple Features for R: Standardized Support for Spatial Vector Data. The R Journal. 2018;10(1):439-46.

10. Massicotte P, South A. rnaturalearth: World Map Data from Natural Earth. 2023.

11. South A. rnaturalearthdata: World Vector Map Data from Natural Earth Used in 'rnaturalearth'. 2017.

12. Bivand R, Rundel C. rgeos: Interface to Geometry Engine - Open Source ('GEOS'). 2023.
